# Dance-based avenues to advance nonpharmacologic treatment of chemotherapy effects (DAANCE): Study protocol for a multi-center, randomized controlled trial

**DOI:** 10.21203/rs.3.rs-6814353/v1

**Published:** 2025-07-07

**Authors:** Bhillie Luciani, Martha Carlson, Gretchen McNally, Madeleine E. Hackney, Jewel E. Crasta, Patrick Schnell, Maryam B. Lustberg, Lise Worthen-Chaudhari

**Affiliations:** The Ohio State University Wexner Medical Center; The Ohio State University Wexner Medical Center; The Ohio State University James Cancer Hospital: The Ohio State University Comprehensive Cancer Center Arthur G James Cancer Hospital and Richard J Solove Research Institute; Emory University School of Medicine; The Ohio State University Wexner Medical Center; The Ohio State University College of Public Health; Yale University Yale Cancer Center; The Ohio State University Wexner Medical Center

**Keywords:** chemotherapy-induced neuropathy, oncology, neurorehabilitation, music, dance, biomechanics

## Abstract

**Background:**

Breast cancer (BC) is among the most common forms of cancer, particularly among women. Chemotherapies that are most effective in treating BC are known to cause chemotherapy-induced neuropathy (CIN), thus leaving survivors with sensory deficits including pain, numbness, and tingling in the extremities; functional deficits such as impaired neuromotor control and motor-cognitive integration; reduced quality of life; and increased fall risk. Current pharmacologic treatments show limited efficacy and create additional unwanted side effects. In contrast, physical activity (PA) has emerged as a promising nonpharmacologic candidate for managing CIN symptoms. The purpose of this trial is to evaluate the effect of Adapted Argentine Tango (Tango) as a potential treatment for CIN. Toward this end, we will assess the intervention effect of Tango vs. the standard of care (SOC) on patient-reported outcomes of sensation, clinically-relevant measures of function, and potential mechanisms of action. We hypothesize that 4 weeks of Tango practice will improve sensation and function more than SOC among BC survivors with CIN and demonstrated balance dysfunction.

**Methods:**

In a multi-center, prospective, randomized controlled clinical trial, participants are randomly assigned (1:1 ratio) to the Tango experimental or the SOC active control arms. Primary outcomes are measured from baseline to after 4 weeks of intervention in patient-reported outcomes (PROs) of sensation and dual-task function. Secondary outcomes include additional PROs, such as fatigue, and clinical measures of interest after 4 and 8 weeks of intervention and 1 month following intervention completion. Exploratory measures include postural control, gait stability, cognitive load, and blood-based biomarker concentrations. Linear mixed models will be used to model changes in PROs and function. The primary estimand will be the difference in mean change in primary outcomes from baseline to week 4 between treatment groups.

**Discussion:**

The scientific premise of this study is that Tango stands to improve CIN symptoms significantly more than the current standard of care by combining PA with auditory-motor entrainment and social engagement. Our findings may lead to a safe non-pharmacologic intervention that improves CIN-related deficits.

**Trial registration:**

This trial was first posted on 12/27/24 at ClinicalTrials.gov under the identifier NCT06749210.

## Introduction

### Background and rationale

Breast cancer (BC) is one of the most diagnosed cancers, with an estimated 2.3 million diagnoses in 2020 that disproportionately affected women (1). Of those that receive a BC diagnosis, it is estimated that 64.5% will undergo chemotherapy treatments with taxane-based agents (2) that cause secondary conditions that complicate aging, such as chemotherapy-induced neuropathy (CIN) (3–9). Up to 80% of breast cancer survivors who undergo chemotherapy develop CIN (4, 8). The manifestation of CIN may cause delay or discontinuation of life-saving chemotherapy treatment and decrease quality of life (8, 10). Symptoms include disabling pain, numbness, tingling, and/or burning in their hands and feet (6, 11, 12); a decrease in neuromotor control (13–16); deficits in motor-cognitive integration (17); and increased fall risk (18, 19). At present, treatment options for CIN are limited (6). Despite extensive research, no pharmacologic intervention has significantly improved the neuromotor, functional, and patient-reported symptoms of chronic CIN among BC survivors. Instead, pharmacologic interventions focus on managing symptom development through nerve pain relief (e.g. gabapentin) or serotonin reuptake inhibitors (e.g. duloxetine). However, these pharmacologic solutions come with side effects, such as drowsiness and suicidal ideology. Therefore, there is a critical need to address the neurosensory motor symptoms of neuropathy created through chemotherapy treatments among BC survivors.

Physical activity (PA) is a promising nonpharmacologic candidate in the treatment of CIN (20–25). Possible training effects of PA include reduced systemic inflammation (26), axonal regeneration in the peripheral nervous system (27), and improved executive function (28). However, additional research is needed for progressive and prescriptive exercises relevant to the resolution and/or reduction of CIN as well as to understand adherence to a program. Social dance has been proposed as a candidate PA to promote adherence (29). Social dance can be delivered as progressively challenging PA (30) that addresses functional targets of CIN such as balance and motor-cognitive integration (17, 31–33), and promotes intrinsic motivation and adherence over conventional exercise programs (17, 29, 31, 34).

Among potential social dance forms, Adapted Argentine Tango (Tango) has been used to improve motor and cognitive impairments in Parkinson’s Disease (32), another population that experiences neuropathy and increased fall risk. As a partnered dance, Tango is a form of PA with potential to active the human dynamic system via rhythmic entrainment (17, 29, 35), has been associated with better intrinsic motivation to participate than traditional exercise programs (29), and may serve as rehabilitation for postural control (36–38), gait function (36, 39), and patient-reported outcomes of symptoms similar to those reported by BC survivors with CIN (40–42). Further, pilot data show the feasibility of Tango as a potential PA-based solution for cancer survivors with CIN symptoms (17, 29, 38).

We hypothesize that Tango will improve CIN compared to usual care. To evaluate this hypothesis, we will evaluate the effect of Tango, a light to moderate intensity social dance adapted for persons with mobility deficits (30, 32) and breast cancer survivors (17), on patient-reported symptoms of chronic CIN, function with respect to motor-cognitive integration, and potential physiological mediators of sensation and function among BC survivors. Research by the multiple principal investigators (MPIs) established Tango as feasible for aging survivors (up to 82 years old) to engage in biweekly at a dose of 33(4) (mean(SD)) min/session with high satisfaction and positive effects (32, 38) and specifically efficacious for motor-cognitive integration in doses of approximately 20 min/class (17). We hypothesize that Tango will improve sensation, dual-task function, and their potential physiological mediators among BC survivors who demonstrate CIN with balance dysfunction.

### Objectives

The primary objective of this experiment is to rigorously evaluate Tango as a nonpharmacologic intervention for neuropathy symptom relief – including patient-reported numbness and tingling (Aim 1 primary outcome) and dual-task function (Aim 2 primary outcome) - and to establish the first exploratory data regarding mechanisms supporting positive effects, such as change in brain activity, biomechanics, and blood-based biomarkers. We hypothesize that 4 weeks of Tango practice (2x/week; 15–30 min dose of movement-to-music per session) will improve sensation (Aim 1) and dual-task function (Aim 2) more than usual care among BC survivors with CIN and demonstrated balance dysfunction; we further hypothesize that exploratory measures will show change with 8 weeks of Tango practice.

## Methods

### Study Design

**Figure 1:**
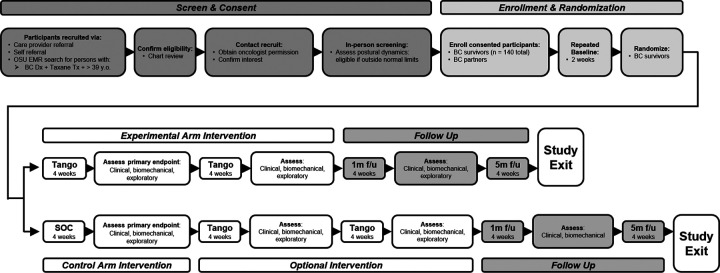
Flow chart of study design from screening through the follow-up (f/u) period with assessments indicated throughout. Postural control data will be taken at each Tango session in addition to all assessment periods. Each assessment timepoint may contain 1) clinical measures: PROs, dual-task function, and upper extremity function; 2) biomechanical measures: postural control and gait stability; and 3) exploratory measures: biomarker and EEG sampling. For more detailed information, refer to tables 1 & 2. Processing of data will happen after the conclusion of the follow-up period per participant. Analysis of the primary outcome measures will be conducted after all data has been collected.

This is a multi-center, prospective randomized controlled study of superiority between an experimental intervention and usual care control arm. Eligible participants who provide informed consent will be randomly assigned to the Tango experimental (EXP) or the standard of care (SOC) control group (CON) in a 1:1 ratio. The study primary endpoints are changes from baseline to 4 weeks of intervention in the patient-reported outcome (PRO) of the sensations of numbness and/or tingling (Aim 1) and the quantitative outcome of dual task function during performance of a cognitive challenge simultaneously with a walking task (Aim 2). As an exploratory endpoint, we will assess the effect of Tango on the blood-based biomarkers that potentially mediate sensation and function as well as on cognitive load during a dual-task paradigm as measured by brain activity (i.e. high gamma) (Aim 3). Tables 1 & 2 present the data collection schedule for the EXP and CON groups, formatted per the Standard Protocol Items: Recommendations for Interventions Trials (SPIRIT) recommendations (43–45). Figure 1 illustrates the overall study design as a flow diagram. As detailed in Tables 1 & 2, outcomes are assessed at the following timepoints: repeated baseline (Baseline), primary endpoint (4 weeks of intervention or SOC), 8 weeks of intervention, 12 weeks (follow-up after a 4-week period of no structured intervention being offered), and weekly for 6 months following intervention end. Secondary outcome measures include clinical and biomechanical tests of function and patient-reported outcomes that complement the primary outcome measures in addition to falls incidence. Tertiary outcome measures, listed in Table 3, include within-session effects, satisfaction collected at session end, and surveys used to inform monitoring and shaping of interventions including Rating of Perceived Exertion - Physical (RPE-P; 6–20 scale), Rating of Perceived Exertion - Mental (RPE-M; 6–20 scale), and Intrinsic Motivation Inventory (IMI). To support beneficence, individuals randomized to the control arm will be given the option of participating in the experimental intervention after primary outcomes measurement.

#### Repeated baseline schedule

Repeated baseline data have previously been collected and analyzed as described in Lantis et al., 2023 (46). Select measures (Tables 1 & 2, indicated with x^†^) are collected repeatedly prior to intervention to characterize within-subject variability (WSV) in CIN-related symptoms and function at baseline.

Similar to Lantis et al., 2023 (46), repeated baseline measures for postural control were achieved through collection of the postural control dynamics during silence (QEC) at the beginning of up to 3 visits, including the first screening test of postural control and 3–4 additional days.

### Study setting

The study is currently being conducted at The Ohio State University (OSU) in Columbus, Ohio (OH) and Yale Cancer Center in New Haven, Connecticut (CT). The research protocol has been approved by the OSU Institutional Review Board (IRB) and Yale has ceded review to this board as the single IRB for the multisite clinical trial. At OSU, participants are recruited from the Stephanie Spielman Comprehensive Breast Center (SSCBC) oncology practice as well as from the greater central OH community. At Yale, participants are recruited from the Yale Center for Breast Cancer (CBC) within the Smilow Cancer Center oncology hospital network as well as from the greater south central, CT and northern New York City, New York communities. Consent is obtained in a quiet and private setting prior to research activity either electronically, via REDCap (Research Electronic Data Capture, Nashville, TN), or using a paper version which is stored securely in a locked cabinet within a secure research-dedicated space at the host institutions. Assessments (i.e., screening, repeated baseline, midpoint, post intervention, and 1-month follow-up assessments) are performed in an outpatient care clinic setting or within the volunteer’s home environment, as preferred by the survivor. The EXP intervention is performed in a group setting of no more than 15 survivors at a time with their invited partners. The SOC portion of the CON intervention is performed by participants in their own home environment. Participants are compensated $20 per in-person assessment session that does not involve interventional instruction, with an additional $20 compensated for sessions that require sensors to be applied to the skin (potential total of up to $280 for participation in all assessments).

### Eligibility criteria

The eligibility criteria of this study follows the criteria described in Lantis et al. (2023) (46). Briefly, participants are eligible for this study if they are 40 years old or older, diagnosed with BC (all stages), experiencing CIN (European Organization for Research and Treatment of Cancer, Chemotherapy-Induced Peripheral Neuropathy outcome measure (CIPN-20) sensorimotor score > 1 or similar on an equivalent measure), finished with taxane-based chemotherapy treatment for at least 3 months, able to understand and comply with directions associated with testing and study treatments, and if they demonstrate postural control measurements outside of normative values (47). Participants are excluded from the study if they meet any of the following criteria: pre-existing vestibular disorders, history of motor deficits or neurological disease other than CIN-related, poorly controlled diabetes (HbA1c ≥ 8.0), non-ambulatory or lower extremity amputation (Note: assistive devices allowed), participating in physical or occupational therapy during the study (Note: engagement in additional therapies or physical activities (e.g. acupuncture) will be documented), or contraindicated to participate in unsupervised activity due to other issues (e.g. herniated vertebral disc).

We expect the study population to be representative of the demographics of the BC survivorship population in the United States between both sites. The CDC reports that 99% of BC cases occur among women and only 1% among men. The CDC census reports the rate of new BC cases in 2019 per 100,000 women by race as White/non-Hispanic 132.5, Black/non-Hispanic 128.4, American Indian and Alaska native/non-Hispanic 101.7, Asian and Pacific Islander/non-Hispanic 104.5, and Hispanic 101.9 (48).

### Recruitment and screening

This research opportunity is posted on public-facing websites including ClinicalTrials.gov (NCT06749210), Research Match, and Study Search to allow volunteers to self-refer by calling a dedicated medical center phone line or emailing the secure address Tango@osumc.edu. Further, advanced practice providers (APPs) refer eligible and interested clients to our research staff to complete screening. The study staff work onsite within the OSU SSCBC and Yale CBC outpatient facilities at least 2 days per week to follow up with volunteers and referring APPs. In addition, queries are run with electronic medical records (EMR) to identify potentially eligible clients of the OSU academic medical center system. Reports are generated using the criteria of a taxane-based chemotherapy plan being entered in the EMR and filtered by age (≥ 40 years) and prescribing physician. Through chart review, we identify the subset of clients who meet the study previously mentioned inclusion criteria. Researchers are provided with a list of potential recruits from a pharmacist on staff with SSCBC and Yale Cancer Center, which includes name, stage of breast cancer, whether they were exposed to taxane-based chemotherapy, and medical record number. Clinical research assistants then pre-screen participants from this list using the previously mentioned criteria.

For clients that meet the inclusion criteria, we request approval from the treating oncologist to contact the client for recruitment purposes. Eligible clients who are approved for recruitment by their oncologist are contacted by phone to inquire whether CIN symptoms persist and, if so, to determine interest and availability in participating in this study. Those able and interested are asked to schedule in-person screening of postural control, which involves attempting a short, but challenging balance task reported to distinguish fallers from non-fallers by Maki et al. (1994) (49). As previously reported (50), study volunteers stand quietly and bilaterally for 30 s on a balance plate (Bertec Corp, Columbus, OH) with eyes closed (QEC). Center of pressure (COP) variables of interest are calculated per Prieto et al. (1996) (51) and Roerdink et al. (2011) (52). Survivors are offered enrollment in the study if they demonstrate QEC postural control function that is outside of the estimated 70% confidence interval (CI) (47) of healthy, age-equivalent normative values in (1) COP ellipse area, (2) medial-lateral variability, (3) medial-lateral velocity or outside of the estimated 95% CI in terms of (4) COP complexity. Values corresponding to these inclusion thresholds are >400, >4.0, >11.0, <0.6, respectively.

### Adequacy of the potential participant pool

Between Yale and OSU, approximately 2300 new BC patients are seen annually (Yale n=1000, OSU n=1300), 500+ of whom will receive taxane-based cytotoxic chemotherapy annually. We conservatively estimate that approximately 50% of BC patients treated with cytotoxic chemotherapy will experience persistent CIN and measurable postural control deficits (Worthen-Chaudhari et al., 2018) yielding an eligible patient pool that grows by at least 113 BC survivors per site, per year. In addition to this annual presentation of new individuals with BC, both sites have established survivorship programs that serve thousands of BC survivors, including survivors not seen within our medical centers, who live with chronic CIN. Given the interest in non-pharmacologic options for survivorship treatment and lack of pharmacologic interventions to treat CIN (12), we anticipate screening 500 eligible individuals per year or 40 per month and enrolling up to 25% (i.e., 10 survivors per month) across sites.

Lastly, attending classes with an invited guest (dance partner) was found to affect engagement, by improving attendance of survivors (38). Therefore, we encourage participants to invite a partner to attend training sessions with them and for those who prefer not to invite from their social circle, we provide partners from a pool of talented university students within the relevant departments (e.g. Dance, Music, Health and Rehabilitation Sciences) at both sites to activate social engagement for enrollees randomized to the Tango experimental intervention.

### Interventions

The EXP intervention occurs over an 8-week period at a frequency of 2x per week with follow-up measures taken at 12 weeks. The CON intervention starts with 4-weeks of SOC and crosses over to experimental for the following 8 weeks at a frequency of 2x per week. No intervention session with accompanying data collection measures will last longer than 1.25 h total.

#### Codesign of Research Study Design and Intervention

Integration of patient lived experience and expertise was one of the key objectives for our group (53). Co-design is a meaningful, participatory collaborative process in research design and includes engagement of patient advocates within all stages of the research design and execution process (53). Early on in designing our interventional study, we reached out to our colleague patient advocate for input on the study planning phase. The PIs discussed the intent of the study and reached a shared collaborative understanding of the research question and how to best structure the clinical trial. Decisions at this stage were influential for all subsequent research processes. One of the central changes that occurred as a part of this discussion was shortening the waiting period from 8 weeks to 4 weeks for patients randomized to usual care. The reason for this change was based on careful deliberation of patient advocate input regarding how long survivors living with neuropathy can be expected to wait to receive potential relief through experimental intervention. Our prior work demonstrated relief from CIN dysfunction within 4 weeks of participation in the experimental intervention, therefore, we set the control wait period and primary outcome evaluation for both arms at 4 weeks (17).

#### Experimental Intervention: Tango

The Tango intervention will consist of 16 Adapted Argentine Tango (Adapted Tango) sessions over 8 weeks, adapted for individuals with mobility deficits by co-investigator (Co-I) Hackney (30,54), further adapted for survivors with BC by lead principal investigator (PI) Worthen-Chaudhari (17), and taught by PI Worthen-Chaudhari. We aim to deliver a dose of skilled movement-to-music of at least 10 min and no more than 35 min per session, with breaks for water and rest offered at least every 10 min. Recommendations for implementation of Adapted Tango as a neurologic intervention focus on prevention of falls, use of implicit learning techniques to convey skilled movement goals, the structure of class (warm-up, lesson, cooldown), scaffolding and shaping lessons over time to establish competency in fundamentals, modifications deemed necessary for specific deficits, and music selection (30,54). Each song used within this intervention is from the tango genre with a single song duration of 1.5 to 8.5 min. Participants may sit or rest as needed during or between songs. Instructors ensure appropriate activity wear choices (e.g., footwear, breast support) and ensure qualified volunteers are present to partner with participants. Instructors complete 16 h of training with Co-I Hackney as well as a certification exam to become qualified to teach the Adapted Tango technique. Volunteers who will partner participants for this study complete Co-I Hackney’s 4 h Balance Management program plus an additional 3 h of training with PI Worthen-Chaudhari during which live instruction skills are evaluated by the PI before volunteers are cleared to partner participants.

The MPIs previously demonstrated that a Tango dose of about 20 minutes of movement-to-music is feasible for cancer survivors with postural control deficits to participate in at a rate of 2 times per week (17). To optimize factors mediating intrinsic motivation, instruction aims to achieve high Enjoyment and Perceived Competence among participants with low Pressure/Tension (55–57) as measured by IMI. We administer the IMI every 2 weeks to assess the achievement of these instructional goals. To assure integrity of the training, Co-I Hackney assists MPIs Worthen-Chaudhari and Lustberg to monitor fidelity of the intervention. This subset of our team – two former professional dancers turned dance scientists and one oncology practitioner – monitor participant symptoms and training progression and troubleshoot any issues that arise through regular virtual meetings.

#### Control intervention with optional 1-way crossover: SOC with EXP

The active control intervention consists of 4 weeks of SOC, as prescribed by each participant’s medical oncologist. Within SOC, medical oncologists may prescribe pharmacologic treatments such as tricyclic antidepressants, anticonvulsants, and serotonin-norepinephrine uptake prohibitors (6,8,58). They may also recommend exercise and/or physical therapy (6). During the 4 weeks, clients are asked, weekly, to document their numbness/tingling due to CIN and any adverse events that occur via a smart phone application (i.e. the MyCap version of REDCap). After completion of outcomes assessments at the primary endpoint of 4 weeks, individuals in the CON arm will have the opportunity to cross over immediately to participate in the EXP tango intervention.

### Procedures/plans to ensure safety of interventions

Procedures to manage pain, functional guarding, inclement weather, seated interventional options and depression and anxiety monitoring are described in Lantis et al. (2023) (46).

### Outcome measure procedures and analysis

#### Primary outcome measures

##### Sensation (Aim 1):

We measure the impact of Tango on sensation using patient-reported outcomes (PROs) of “numbness/tinging/burning” via a validated 11-point Likert scale (59) that queries symptom severity in the prior 7 days. All surveys are administered through OSU’s instance of the REDCap database for in-person visits and through the REDCap phone application (MyCap) for data entry that participants perform remotely (e.g., SOC activity, follow-up period). Surveys are completed by the participants themselves with no data entry from staff. Answers are reviewed manually by authorized research collaborators, who prompt participants to complete or clarify answers as needed and document their responsibility for review from a list of authorized data reviewers.

##### Dual-task function (Aim 2):

We measure function using validated clinical tests, performed in a clinical space in an area that is distraction free. The *Timed Up-and-Go test (TUG)* is a timed test of a person’s ability to stand from a chair, walk 10 feet (3 m), turn around, and return to sitting (60). Presence of CIN has been associated with longer TUG times (61–64). To measure dual-task function, we use the TUG performed simultaneously with a cognitive task consisting of audibly counting backward by subtracting 3 from a given number (*TUG-Cog*). We previously reported that BC survivors with CIN demonstrated dual-task functional deficits with up to 78% demonstrating increased risk of falling as measured by TUG-Cog (17).

#### Secondary and tertiary outcome measures

##### Patient-reported outcome (PRO) survey instruments for measuring symptomatology:

Patient self-report surveys data are collected as described in the *Primary Outcome Measures, Sensation* section above. In addition to the primary PRO-based outcome measure, we collect the following PRO measures:

###### CIN symptoms of temperature.

Self-reported sensations of “hotness” or “coldness” in the last 7 days are collected via a validated 11-point Likert scale (59).

###### European Organization for Research and Treatment of Cancer’s Quality of Life Questionnaire, Chemotherapy-Induced Peripheral Neuropathy (CIPN-20):

is a validated 20-item patient reported questionnaire instrument for longitudinal evaluation of neuropathy symptoms induced by chemotherapy for a recall period of “during the past week” (65).

###### Other symptoms.

Using the Patient-Reported Outcome version of the Common Terminology Criteria for Adverse Events (PRO-CTCAE) items 39 through 56, cancer-related symptoms such as balance problems, nausea or vomiting, dizziness, sensitivity to light or noise, feeling like “in a fog”, confusion, sadness, and anxiety can be queried for the recall period of the past 7 days (66).

*The Brief Pain Inventory (BPI)* is validated to elicit momentary (“right now”) and retrospective (prior 24 h period) self-reported pain and relevant functional capacity (67).

*The Brief Fatigue Inventory (BFI)* is validated to elicit momentary (“right now”) and retrospective (prior 24 h period) self-reported fatigue and relevant functional capacity (68).

*The Short Form Health Survey (SF-36)* is a quality of life (QOL) measure in cancer survivorship validated to elicit momentary and retrospective (last 4 weeks) assessment of an individuals health status. One report identified this survey as a predictive tool for assessing fall risk amongst cancer survivors (69).

*The Generalized Anxiety Disorder 2-Item (GAD-2)* is a validated assessment of retrospective (last 2 weeks) self-reported anxiety (70).

*The Patient Health Questionnaire-2 (PHQ-2)* is a validated assessment of retrospective (last 2 weeks) self-reported depression (Li et al., 2007).

###### Ecological momentary assessment (EMA).

EMA is a method of experience sampling that captures an individual’s self-report of their symptoms “right now” (72). We modified self-report assessments as needed to elicit self-report of symptoms “right now” at the beginning and end of intervention sessions as well as remotely through the MyCap phone application. Toward this end we used EMA items from the BPI and BFI and created EMA versions of these surveys: 11-pt Likert scale assessment of numbness and tingling in the extremities, 11-pt Likert scale of hotness and coldness in the extremities and the EORTC CIPN-20. Rather than modifying the PRO-CTCAE items 39 to 56, we used the Sports Concussion Assessment Tool symptom EMA survey of physical, cognitive, and emotional symptoms of mild brain injury which align with PRO-CTCAE items 39 to 56.

###### Assessment during the activity just performed:

Finally, to query whether symptomatology was affected by the interventional activity, we modified all PRO instruments to query for the recall period of “during the activity just performed”.

*Satisfaction with intervention* is measured after each class using a 7pt Likert scale and prompt for feedback about what did/did not work per class. Feedback is used to improve future sessions.

*The Intrinsic Motivation Inventory (IMI)* was developed from the perspective of Self Determination Theory to assess 7 dimensions of experience: Interest/Enjoyment, Perceived Competence, Effort/Importance, Pressure/Tension, and Choice (73). We administer the 9-item short form of the IMI monthly to optimize instruction around low Pressure/Tension and high Interest/Enjoyment (56,57) as well as to explore relationships between adherence, motor effects, and IMI dimensions including perceived benefit (i.e., Effort/Importance).

##### Function

###### Activity tracking:

Participants are asked if they have participated in the following activities: physical therapy, occupational therapy, fitness activity, other therapeutic or fitness activities. If any category of activity is checked as having occurred then participants are prompted to estimate the amount of time spent doing the activity since we last saw them (hours, minutes). If participants indicate that physical or occupational therapy were started during the study period, we remind them that these are an exclusion criterion and give them the choice of postponing engagement in skilled therapy until after completion of the study or termination of study participation.

###### Falls tracking:

Falling is defined as an unexpected loss of balance in which an individual comes to rest at a position lower than before the unexpected event (74). We elicit self-report of falls and loss of balance using the question “How many times have you fallen or felt like you lost your balance since we last saw you?” Responses are typed by the participant and reviewed for incidence of falls, incidence of loss of balance, and details offered about either incidence. During the 6-month follow-up period after intervention end, each participant will use the MyCap application to report falls weekly.

###### Barriers to participation:

Participants are asked “To help us understand barriers to participation, if you missed a session, please indicate the reason why you were unable to attend (e.g., schedule conflict, transportation, didn’t feel up to it, forgot).”

##### Clinical

To measure *dynamic balance function*, we collect Mini Balance Evaluation System Test (MiniBEST), which evaluates sensory organization, anticipatory and reactive postural control, and dynamic gait indices (75) was found to discriminate BC survivors from controls in at least one prior study (64), and has been recommended for use in studies of neuropathy (18). The subtasks enable calculation of postural control variables of interest for predicting fall risk.

To measure *walking endurance*, we collect the 6-minute walk test (6mwt), which quantifies the distance an individual can walk within 6 minutes, where shorter distances correlate with poorer function including among survivors with CIN (76). Meta-analysis of 6mwt results among adults with pathology or fear of falling indicate that 14.0–30.5 m represents the minimum clinically-important difference (MCID) for improvement in this measure (77). Among BC survivors, a separate meta-analysis found that BC survivors cover less distance as compared to individuals living without cancer (78). The 6mwt is performed in a designated space within each site’s oncology clinic, in a quiet area without distractions. Participants walk in a loop with the following configuration: a straight distance of 19.8 m minimum marked by tape on the floor that participants turn around to their left; participants are cued to walk straight between tape marks and to turn comfortably at the end of each straightaway at the fastest pace they feel they can maintain for 6 min, resting as needed in a standing position before continuing.

As a further measure of CIN and sensation, we will use the *Total Neuropathy Score (TNS) - Revised* (79) (TNSr) with the “sense of touch” section added per Streckmann et al. (2024) (80). This tool measures peripheral nerve impairments in the upper and lower extremities by evaluating symptoms, sensation, and deep tendon reflexes. In the shortened and revised TNS, subjective sensory symptom presence of tingling, numbness, and pain are evaluated in both the upper and lower extremity. Sensation of vibration is evaluated with a Rydel-Seiffert 128 Hz tuning fork on the bony processes of the upper and lower extremities from distal to proximal, while perception of touch is evaluated by stroking the upper and lower extremities. Finally, deep tendon reflexes of the upper and lower extremities are assessed from distal to proximal.

*Upper extremity (UE) function* will be measured using the back scratch item from the Senior Fitness Test (81). Grip strength will be measured using a standardized digital hand grip dynamometer (Jamar 3-piece Dynamometer, Fabrication Enterprises, Elmsford, NY). In the rehabilitation of individuals with neuropathy, grip strength has shown to be an important clinical outcome in assessing upper extremity impairment and is an important predictor of functional status, muscular endurance, and overall strength (82–84). Participants will be seated comfortably with their elbow flexed at 90 degrees, forearm in a neutral position, and wrist in slight extension. After a demonstration and one practice attempt, each participant will perform three maximal grip efforts with a rest period of 10 seconds between trials to prevent fatigue. The maximum of the 3 trials will be used for analysis.

###### Occupational performance:

The Canadian Occupational Performance Measure (COPM) (85) will be administered through a collaborative process with each participant to identify the patient-reported 5 most important occupations related to self-care, leisure, or productivity. In a semi-structured interview with the study therapist, participants will rate their current performance and satisfaction with performance on each activity using an ordinal scale ranging from 1 to 10 points, with higher scores indicating better scores. These assessments will be conducted at baseline (pre-intervention) and repeated at the conclusion of the intervention (post-intervention) to gauge any changes in functional performance and perceived satisfaction. A change of 2 points is considered the threshold for a clinically meaningful change (85). This individualized, client-centered approach of this COPM instrument allows for precise measurement of intervention outcomes, ensuring that improvements in daily functioning are accurately captured and meaningful to the participants (86–88).

##### Biomechanical

Measures of *walking variability and stability* have been found indicative of mild cognitive impairment (89), age (90), fall risk (91–93), and neuropathy (94,95) regardless of age or type of cancer (96) including specifically in BC (16,94,97). Gait stability measures will be collected using inertial measurement units (IMUs) (Trigno Centro, Delsys, Natick, MA) affixed to the foot, leg, thigh, pelvis, and/or lower cervical spine during while participants perform the 6mwt (98), where foot IMU sensors will detect heel strike and toe off events within the gait cycle. The non-linear measures of local dynamic stability (99–102) and recurrent quantification analysis (RQA) (103) will also be analyzed. An IMU sensor placed on the upper torso (C7 vertebral spine) will collect gyroscopic data to detect bouts of steady-state walking behavior along a straight path within the 6mwt. Steady-state bouts of walking that are longer than 4 strides (101,104) will be used to calculate non-linear measures of gait stability. From this, mean Lyapanov exponent and RQA measures will be calculated over the course of the 6mwt. From the steady-state bouts, the coefficient of variation (CV) will also be calculated for step length, gait speed, and other measures of variability previously found sensitive to health status (91,92,105).

We measure *postural control* through center of pressure (COP) data collected in accordance with our group’s previous studies of balance deficits (46,47,50). Data are recorded on a portable balance plate (Bertec Corporation, Columbus, OH) at while participants stand on the plate quietly with their eyes closed (QEC). The data are captured using custom software written in LabVIEW (National Instruments, Austin, TX) at a sampling frequency of 1000 Hz and consist of one channel of vertical force data (Fz) and two channels of moment data (Mx, My) over time.

Additionally, we complete the QEC measure while listening to music (QECm) to examine the relationship between music listening and postural control over the course of the intervention, as it has been shown that postural control improved amongst healthy college-aged individuals after listening to music (37). We follow the same protocol listed in our group’s previous work, where a participant completes a QEC posture while listening to the musical composition “La Cumparsita” (46).

From the QEC and QECm data, we calculate the COP time series for the 30-s duration of QEC condition performance. We then calculate the measures of future fall risk from the two tasks: COP variability in resultant and medial– lateral planes (RMSr, RMSml), medial-lateral sway velocity (COPv), and 95% ellipse area (COPa) for the 30-s duration per Prieto et al., (1996) (51) well as sample entropy using the increment method of calculation (SEI) (52).

*Co-contraction index (CCI)* is the ratio of the muscle activity produced in the agonist versus the antagonist muscles of the leg, measured via electromyography (EMG), which can provide unique insights into neuropathy effects (106). For this protocol, we measure signals in the tibialis anterior, lateral gastrocnemius, and soleus muscles during performance of the 6mwt, QEC, and QECm tasks.

### Exploratory measures

#### Blood-based biomarkers

Blood sample data collections will occur at baseline, after 4 weeks of usual care for members of the CON group, and after 8 weeks of intervention for members of the EXP group. Blood sample collections will occur in the same facilities mentioned in the *Study Setting* section, during scheduled clinical or research appointments. Blood will be drawn in two 6 mL EDTA tubes at the timepoints described previously. Best practice regarding collection and centrifugation will be followed to extract the plasma layer and store aliquots (107). All cryovials containing plasma will be labeled and stored in a −80°C freezer, accessible to laboratory personnel only. Aliquots from each timepoint per participant ID will be shipped to Yale facilities for further processing on an annual basis.

Blood-based biomarkers of interest include targeted indices linked to breast cancer (7,108), neurorecovery (109–111), and neurotoxicity/trauma (7,112). Panels of markers will be measured in plasma. We will investigate a battery of biomarkers using singleplex assays. We list these in order of priority for analysis below in the even that unexpected funding changes impact biomarker analysis budget:

1. Neurofilament light (NfL) and glial fibrillary acidic protein (GFAP) (Benashley et al., 2022; Burgess et al., 2022; Rodwin et al., 2022).
2. Interleukin-6 (IL-6), IL-10, brain-derived neurotropic factor (BDNF) (7,114), tumor necrosis factor α (TNF-α) (109,111).
3. Other cytokine and inflammatory biomarkers associated with neurotoxicity including but not limited to: IL-7, IL-8, IL-9, interferon gamma (IFN-γ), interferon induced protein 10 (IP-10; CXCL-10), macrophage derived chemokine (MDC), monocyte chemoattractant protein 1 (MCP1; CCL-2), transforming growth factor-α (TGF-α), serum amyloid protein (SAA), endothelial markers (ICAM-1, VCAM-1) (108,109).

At the baseline, after 4 weeks for the CON group, and after 8 weeks for the EXP group, we will measure NfL, a neuronal cytoskeletal protein that has indicated axonal damage in animal CIN models and survivors (7). NfL serum concentration will be assessed by ultrasensitive single molecule array (Simoa NF-Light^®^ assay, Quanterix, Billerica, MA).

We will investigate synaptic plasticity through via BDNF and GFAP, sampled at baseline, after 4 weeks for the CON group, and after 8 weeks for the EXP group. BDNF levels will be analyzed at the Yale facility, while GFAP levels will be assessed by the Quanterix laboratory (Quanterix, Billerica, MA).

#### Cognitive load

Brain activity will be collected via electroencephalography (EEG) and occur at baseline, after 4 weeks of SOC or INT, and after 8 weeks of intervention. EEG data collection will occur in the same facilities mentioned in the *Study Setting* section, using a mobile EEG system (eego, ANT Neuro, Netherlands) with 24-channels (waveguard net, ANT Neuro, Netherlands), sampling at a frequency of 2048 Hz. EEG signal processing and analysis will follow Crasta et al. (2018) (115) using BV Analyzer (Brain Vision LLC, Garner, NC) to extract mean activity per frequency band, phase synchrony, and event-related potentials.

The protocol to assess cognitive load follows that of Worthen-Chaudhari et el., 2024 (17). EEG signals were collected during the performance of the following postural and motor-cognitive tasks. Conditions listed in Table 4 below are completed by each participant while standing with their shoes off, feet 7 cm apart from the inside of their feet, hands to their side, and facing comfortably straight ahead. Participants will be guarded by trained research staff during standing tasks and will get a break at the halfway mark of each task. Each task lasts for 3 minutes. Auditory stimuli will be presented through ER-3A-inserted earphones (Etymotic Research, Elk Grove Village, IL) via a custom-made program in PsychoPy (Open Science Tools, Nottingham, England).

For the visual and auditory oddball, each stimulus is presented for 500 ms with a random inter-stimulus interval from 500 ms to 1 second. A total of 100 stimuli are presented (80 standard and 20 oddball).

#### Within-session effect

At the beginning and end of each in-person intervention session, we collect a subset of PRO data with which to evaluate the effect of the session (Table 3). Each PRO measurement is collected from participants using a computer, a touch screen tablet, or a cell phone.

### Power calculations

Allowing for up to approximately 30% loss to follow-up, we plan to enroll 70 subjects per arm to retain 50 per arm per site. This drop-out rate is conservative, corresponding to criteria that we used previously to assess an intervention’s feasibility (38,116). We use a two-sample t-test 5% significance level (two-sided) as a conservative approximation to our analysis procedure. Due to additional covariate information and early information from subjects lost to follow-up, we expect greater power from our linear mixed model analysis.

To calculate effect size of the Aim 1 primary outcome measure: CIN sensation, we used the Numbness and Tingling (NT) 11-point scale (i.e., 0–10) from a study of physical activity for CIN among MBC survivors that observed post-chemotherapy scores with a standard deviation of 2.5 in the control arm and 1.9 in the treatment arm (Kleckner et al., 2018). Using the same standard deviations we have approximately 80% power to detect a minimum clinically significant difference of 1.25 (corresponding to minimally clinically important difference of 2.5 points on the 0–20 CIN-20 5-item sensory scale).

To calculate effect size of the Aim 2 primary outcome measure: dual-task function in the motor and cognitive domains, we used an effect size of 0.56 (Cohen’s *d*) based on preliminary data from 14 BC survivors with CIN participating in our pilot work (R21-AG068831); the effect size was verified as a conservative estimate as compared to a later calculation using a larger cohort of 46 BC survivors with CIN (17). Enrolling 70 participants per group (140 total) allows for a drop-out rate of up to 30% to yield full follow-up on 50 participants per arm and 80% power to detect the target effect size at the 5% significance level. This drop-out rate is conservative, corresponding to the criteria that we previously used to assess an intervention’s feasibility (38,116).

### Randomization

After a participant has qualified, consented to participate in this trial, and completed repeated baseline testing, researchers randomize them 1:1 into one of two groups: EXP or CON. Randomization is blocked and stratified by site, age (≥61 vs <61), baseline CIN sensation (Likert ≥4 vs <4), and baseline functional status (TUG-Cog ≥12.64 s vs <12.64 s) based on the results of previous work in our lab (17). CIN sensation was identified through the 11-point Likert scale (59) administered during the repeated baseline period. Functional status is identified by averaging the times of 3 TUG-Cog trials that participants completed during the repeated baseline period.

Randomization is accomplished using a schedule that was generated by the statistician prior to the trial opening to enrollment. The clinical research coordinator will assign allocation and enroll participants. One member of the research team is blinded to the randomization schedule prior to allocation; this researcher administers TUG and TUG-Cog to participants throughout the study. Study participants cannot be blinded; they will inevitably know their group assignment as they are participating in a distinct intervention in comparison to usual care.

### Data management

Each study participant is assigned a unique six-digit identification number that cannot be traced to their protected health information. Participant data, coded using these identification numbers, are stored in a central database using REDCap. REDCap is a secure web-based platform that is designed for clinical trials which meets both HIPAA and 21 CFR. Clinical and biomechanical data are collected by study staff who input results into REDCap manually or via data upload. PRO data is manually entered into REDCap by study participants: participants undergoing the Tango intervention fill out questionnaires within REDCap before the start and at the end of each Tango session. SOC participants fill out questionnaires within MyCap, a mobile device application of REDCap once per week during the SOC wit period, for the purpose of adverse events reporting and symptoms tracking, then transition into the Tango intervention protocol in which they fill out questionnaires within REDCap before the start and end of each Tango session.

### Statistical analysis

All outcome analyses will be performed on an intent-to-treat basis, and all hypothesis tests will be two-sided and at the 5% significance level or 95% confidence level. Longitudinal linear mixed models will be used to model changes in PROs and dual-task function. Fixed effects will be included for condition, session number within condition, baseline outcome measure, age, diabetes status, physical fitness activity (defined by weekly duration and metabolic equivalents), and number of previous chemotherapy lines. Random effects for subjects and study center will be used to account for within-subject and within-center correlation, and covariance estimated by restricted maximum likelihood. Exploratory Aim 3 will be assessed via causal mediation analysis using marginal structural models. The primary estimand will be the contrast representing the difference in mean change in outcome measure from baseline to week 4 (end of randomized treatment comparison) between treatment groups.

While we expect balance between groups due to randomization, we will adjust for potential confounders including age, body mass index, concurrent medications, and other variables if an imbalance between groups occurs by chance. Pearson’s *R* will be calculated to correlate PROs, functional outcomes, biomechanical outcomes, brain activity, and biomarker outcomes. We use a longitudinal mixed effect model to analyze all outcomes and do not impute missing outcomes. Subjects will be analyzed in the group to which they were randomized regardless of compliance with the assigned intervention. We will evaluate the effect of the number of intervention sessions completed on outcomes.

### Trial monitoring

No regular external trial auditing is scheduled. However, trial monitoring personnel include a safety officer (SO) appointed by the funding body (NIH) and the two PIs, Worthen-Chaudhari (PI) and Lustberg (MPI). Study staff and the PI will review information regarding safety, data quality, and validity on a weekly basis, informing the MPI immediately of any concerns. All data related to recruitment, screening, enrollment, baseline measurement, and intervention measurement will be reviewed weekly by the PI. Data collected from enrolled participants will be reviewed weekly by the statistician with the PI. Additional investigators and study staff will be asked for their input or expertise as needs arise. If unforeseen hazards or risks are identified that may lead to serious adverse events, the PI consults the appropriate members of the team, including the NIH-appointed SO.

### Adverse event monitoring and reporting

Participants randomized to the CON group are asked to report adverse events at the beginning of each week during the SOC period using the MyCap application, a mobile application version of REDCap that participants install on their smart phone devices. Once these participants engage in the intervention (one-way crossover period) they report adverse events on the same schedule as the EXP group. Participants randomized to the EXP arm complete adverse event reporting each time we see them (2x per week) via REDCap. With respect to the adverse event of falls, participants are asked about the incidence of falls and loss of balance. Researchers review REDCap responses once per week for the CON participants within the SOC period and prior to the start of Tango lessons for participants engaging in the Tango intervention (EXP and crossed over CON). If an adverse event is reported, the PI is notified. Classification of the events as Serious or not occurs within 24 hours.

#### Serious adverse events

All serious adverse events are immediately reported to the research team and PI and then reported to the IRB, sponsor, and SO within 24 h.

#### Nonserious adverse events

Any nonserious adverse events are reported to the SO in biannual meetings and are subject to review prior to receiving authorization for the continuation of the research.

#### Biannual reports

A biannual open report summarizing study progress and safety monitoring data is reviewed by the SO and representatives from the NIA/NIA. Approval of the SO is required for the trial to continue.

#### Post-trial care

Both Tango and SOC participants continue to report adverse events and CIPN EMA for 6 months post-intervention. Researchers monitor responses and check in with participants as needed. Upon completion of the 6-month follow-up period researchers communicate their appreciation for participation.

### Protocol amendments

Any changes to the protocol require written amendments that must be approved by the NIH and IRB. Upon acceptance from the sponsor and IRB, the PI makes updates to the study record published on ClinicalTrials.gov. If the PI determines that a protocol deviation is necessary for safety reasons, scheduling, recruitment, or personal accommodations for participants, the IRB will be notified immediately.

### Confidentiality

Any physical documentation containing protected health information is stored in a locked cabinet located within research or clinical designated space that is locked and/or monitored when not occupied. Digital documentation is stored in REDCap and/or on secure servers requiring password authentication that are behind secure firewalls.

### Access to data

Study staff, OSU IRB, and representatives of the NIH have access to study data. All those who have access to study data are trained in HIPAA standards for privacy protection and do not refer to confidential information with anyone outside of the study team.

### Dissemination policy

The results of our research will be disseminated to (a) the scientific community; (b) breast cancer survivors; (c) persons with symptoms of neuropathy; (d) participants of the trial who wish to view their data after the 5-year study is complete; and (e) the public. The results of this research will be presented at scientific conferences, including the Multinational Association for Supportive Care in Cancer (MASCC), American Congress of Rehabilitation Medicine (ACRM), and American Society of Biomechanics (ASB). Additionally, results will be published in peer-reviewed journals.

## Discussion

As BC survival rates improve due to available treatments such as chemotherapy, the long-term quality of life for survivors has become a crticial focus of care. The rationale for this study stems from accumulating evidence that PA represents a viable non-pharmacologic avenue to treat one debilitating symptom of survivorship: chronic CIN (6, 17, 25). As a candidate form of neurologic dance training, Tango has been demonstrated to improve quality of life in populations with neuropathology (38, 46, 54). The scientific premise of this study is that Tango stands to achieve more gains than the current medical SOC for CIN by combining PA with rhythmic musical engagement and delivering the intervention in a socially-engaged environment. Based on previous data establishing safety, feasibility, and initial effect of Tango for survivors with CIN (38, 46), we have designed a randomized controlled trial to compare the effectiveness of the SOC versus an experimental arm that delivers Tango. We assess comparative effectiveness in terms of patient-reported, functional, and neurophysiologic outcomes relevant to BC survivorship. Our findings may lead to a safe, effective, simple, economical, non-pharmacologic intervention that improves CIN-related deficits and symptoms through activity that can be performed with a friend or loved one. Adding small doses of NDT as standard of care (SOC) for survivors with CIN is a simple, cost-effective solution that can be implemented anywhere in the world without major regulatory hurdles. Better functional recovery for survivors with CIN will lead to improved quality of life, short-term, and long-term health and wellness outcomes for these individuals. Therefore, the risks that participants in this study might incur are minor relative to the potential benefits of improving symptoms and function for BC survivors with CIN and measurable balance deficits.

### Trial status

This study has been active and open for enrollment since January 17th, 2025. Enrollment is expected to be completed by Dec 2028. Intervention delivery and follow-up are expected to be completed by July 1st, 2029. The clinical trial number associated with this trial is NCT06749210.

## Supplementary Material

Supplementary Files

This is a list of supplementary files associated with this preprint. Click to download.

- DAANCEOSUConsentHIPAASurvivor14.pdf
- DAANCESPIRITchecklist.docx

## Figures and Tables

**Table 1:** SPIRIT report of intervention effect outcome measures for EXP group.

	Study Period						
	Enrollment		Allocation	Post-Allocation			
Timepoints	3 + months post-last taxane exposure	Baseline	0	EXP 4 weeks (Tango midpoint)	EXP 8 weeks (Tango end)	EXP 12 weeks (1-month follow up)	6-month follow up period, assessed weekly
ENROLLMENT:							
Eligibility screen	x						
Informed consent	x						
Allocation			x				
ASSESSMENTS:							
Adverse events (including falls)		x^†^		x	x	x	x
Neuropathy (primary outcome Aim 1)		x^†^		x	x	x	x
TUG-Cog (primary outcome Aim 2)		x		x	x	x	
CTCAE		x^†^		x	x	x	
Pain		x^†^		x	x	x	
Fatigue		x^†^		x	x	x	
GAD-2		x		x	x	x	
PHQ-2		x		x	x	x	
SF-36		x		x	x	x	
Satisfaction with intervention				x	x		
Activity outside of study tracking		x^†^		x	x	x	x
Intrinsic motivation				x	x		
Postural control during silence (QEC)		x^†^		x	x	x	
Postural control during music listening (QECm)		x^†^		x	x	x	
TUG		x		x	x	x	
TNSr+		x		x	x	x	
MiniBEST		x			x		
6mwt (EMG, IMUs)		x			x		
Upper extremity function		x		x	x	x	
Goal attainment (COPM)		x			x	x	
Blood-based biomarkers		x			x		
Cognitive load (EEG)		x		x	x	x	

† indicates a measure was repeated 3 times

**Table 2:** SPIRIT report of intervention effect outcome measures for CON group.

	Study Period							
	Enrollment		Allocation	Post-Allocation				
Timepoints	3 + months post-last taxane exposure	Baseline	0	CON 4 weeks (post-SOC)	CON 8 weeks (Tango midpoint)	CON 12 weeks (Tango end)	CON 16 weeks (1-month follow up)	6-month follow-up period, assessed weekly
ENROLLMENT:								
Eligibility screen	x							
Informed consent	x							
Allocation			x					
ASSESSMENTS:								
Adverse events (including falls)		x^†^		x	x	x	x	x
Neuropathy (primary outcome Aim 1)		x^†^		x	x	x	x	x
TUG-Cog (primary outcome Aim 2)		x		x	x	x	x	
CTCAE		x^†^		x	x	x	x	
Pain		x^†^		x	x	x	x	
Fatigue		x^†^		x	x	x	x	
GAD-2		x		x	x	x	x	
PHQ-2		x		x	x	x	x	
SF-36		x		x	x	x	x	
Satisfaction with intervention					x	x		
Activity outside of study tracking		x^†^		x	x	x	x	x
Intrinsic motivation					x	x		
Postural control during silence (QEC)		x^†^		x	x	x	x	
Postural control during music listening (QECm)		x^†^		x	x	x	x	
TUG		x		x	x	x	x	
TNSr+		x		x	x	x	x	
MiniBEST		x		x		x		
6mwt (EMG, IMUs)		x		x		x		
Upper extremity function		x		x	x	x	x	
Goal attainment (COPM)		x		x	x	x	x	
Blood-based biomarkers		x		x				
Cognitive load (EEG)		x		x	x	x	x	

† indicates a measure was repeated 3 times

**Table 3:** SPIRIT report of within-session assessments. EMA refers to “Ecological Momentary Assessment”

Session Type	Baseline	Tango Intervetion		SOC (remote in MyCap app)
Timepoints	Before session start (Beg)	Before session start (Beg)	After completing session (End)	Weekly
ASSESSMENTS:				
Adverse events	x^a^	x^a^		
Neuropathy retrospective	x^a^	x^a^	x^b^	x^a^
PRO-CTCAE retrospective	x^a^	x^a^		x^a^
Pain retrospective (24 hours)	x	x		
Fatigue retrospective (24 hours)	x	x		
Neuropathy EMA	x	x	x	
CTCAE EMA	x	x	x	
Pain EMA	x	x	x	
Fatigue EMA	x	x	x	
Postural control during silence (QEC)	x	x		
Postural control during music listening (QECm)	x	x		
Activity outside of study tracking	x	x		x^a^
Satisfaction with session activity as neuropathy treatment			x	
Intrinsic motivation			x^c^	
COLLECTED TO FACILITATE TRIAL MONITORING AND INTERVENTION SHAPING:				
Rating of perceived physical exertion (RPE-P)			x^b^	
Rating of perceived mental exertion (RPE-M)			x^b^	

a Recall period = during the last week, since we last saw you, OR since you last answered these questions (whichever is the shortest time period)

b Recall period = during the activity just performed

c Queried every 2 weeks

**Table 4:** EEG conditions

Condition	Description
Seated, eyes open	Resting data collected while seated. Participants will be instructed to watch a fixation point on a screen in front of them.
Quiet standing, eyes open	Participants will be instructed to stand with their eyes open while viewing a fixation point on a screen in front of them.
Quiet standing, eyes closed	Participants will be instructed to stand with their eyes closed.
Standing, eyes open with music	Participants will be instructed to stand while Tango music is played. They will be instructed to watch a fixation point on a screen in front of them.
Standing, eyes closed with music	Participants will be instructed to stand with their eyes closed while Tango music is played.
Visual oddball	While standing, participants will be instructed to mentally count how many odd stimuli (yellow square in the center of the screen) are present while ignoring the repetitive stimuli (red square in the center of the screen).
Auditory oddball	While standing, participants will be instructed to mentally count the oddball tone (1500 Hz) and ignore the standard tone (1000 Hz).

## Data Availability

The datasets used and/or established during the current study will be available from the corresponding author upon reasonable request.

## Abbreviations

6mwt: 6-minute walk test
APP: Advanced practice provider
BC: Breast cancer
BDNF: Brain-derived neurotropic factor
BPI: Brief Pain Index
BFI: Brief Fatigue Index
CBC: Center for Breast Cancer
CCI: Co-contraction index
CCL-2: Monocyte chemoattractant protein 1
CIN: Chemotherapy-induced neuropathy
CIPN-20: Chemotherapy-induced peripheral neuropathy 20-item
CO-I: Co-investigator
CON: Control group
COP: Center of pressure
COPa: Center of pressure area
COPv: Medial-lateral sway velocity
COPM: Canadian Occupational Performance Measure
CT: Connecticut
CV: Coefficient of variation
CXCL-10: Interferon-induced protein 10
EEG: Electroencephalography
EMA: Ecological momentary assessment
EMG: Electromyography
EMR: Electronic medical record
EXP: Experimental group
Fz: Vertical force
GAD-2: Generalized Anxiety Disorder 2-item
GFAP: Glial fibrillary acidic protein
ICAM-1: Endothelial marker – intercellular adhesion molecule 1
IFN-γ: Interferon gamma
IL-#: Interleukin-#
IMI: Intrinsic motivation inventory
IMU: Inertial measurement unit
IP-10: Interferon-induced protein 10
IRB: Institutional Review Board
MCP-1: Monocyte chemoattractant protein 1
MDC: Macrophage-derived chemokine
MIDC: Minimum clinically important difference
NfL: Neurofilament light
MPI: Multiple principal investigators
Mx: Moment data about the x-axis
My: Moment data about the y-axis
NT: Numbness and Tingling Scale
OH: Ohio
OSU: Ohio State University
PA: Physical activity
PHQ-2: Patient Health Questionnaire 2-item
PI: Principal investigator
PRO: Patient-reported outcome
PRO-CTCAE: Patient-Reported Outcome version of the Common Terminology Criteria for Adverse Events
QEC: Quiet eyes closed
QECm: Quiet eyes closed with music
QOL: Quality of life
RMSr: resultant amplitude of postural control data
RMSml: medial-lateral amplitude of postural control data
RPE-M: Rating of perceived exertion – mental
RPE-P: Rating of perceived exertion - physical
SAA: Serum amyloid protein
SCAT: Sports concussion assessment tool
SEI: Sway complexity
SF-36: Short Form Health Survey 36-item
SO: Safety officer
SOC: Standard of care
SPIRIT: Standard Protocol Items: Recommendations for Interventions Trials
SSCBC: Stefanie Spielman Comprehensive Breast Center
Tango: Adapted Argentine Tango
TGF-α: Transforming growth factor-α
TNF-α: Tumor necrosis factor α
TNS: Total Neuropathy Score
TUG: Timed up-and-go
TUG-Cog: Timed up-and-go cognitive
UE: Upper extremity
VCAM-1: Endothelial marker – vascular cell adhesion molecule 1
WSV: Within-subjects variability
